# Understanding Habitats and Environmental Conditions of White-Tailed Deer Population Density and Public Health Data to Aid in Assessing Human Tick-Borne Disease Risk

**DOI:** 10.3390/microorganisms11040865

**Published:** 2023-03-28

**Authors:** Sarah P. Maxwell, Chris Brooks, Pyung Kim, Dohyeong Kim, Connie L. McNeely, Kevin Thomas

**Affiliations:** 1 School of Economic, Political & Policy Sciences, University of Texas at Dallas, Richardson, TX 75080, USA; 2 Laboratory for Human Neurobiology, Boston University School of Medicine, Boston, MA 02118, USA; 3 Center for Science, Technology, and Innovation Policy, George Mason University, Arlington, VA 22201, USA

**Keywords:** tick-borne disease surveillance, Lyme disease, white-tailed deer, wildlife population density

## Abstract

The extent of tick-borne diseases (TBDs) in the United States is largely unknown and underreported. Equitable diagnostic and treatment options may vary by geographic location. Triangulating multi-modal data sources informed by a One Health approach provides robust proxies for human TBD risk. Using data from the Indiana Department of Natural Resources collected from hunters during the white-tailed deer (*Odocoileus virginianus*) hunting season and other sources, we employ a mixed-methods approach based on thematic mapping and mixed effects modelling to determine if deer population density aligns with official disease data at the county level from (1) positive canine serological reports for, anaplasmosis, and Lyme Disease (LD); (2) positive human cases of ehrlichiosis, anaplasmosis, LD, and Spotted Fever rickettsioses; and (3) tick infectivity. We propose the need for multimodal data analysis using a variety of potential proxies to better estimate disease risk and inform public health policy and practice. We find similar spatial distributions between deer population density and human and canine TBDs in northeastern and southern Indiana, which are rural and mixed geographic areas. Overall, LD is more prevalent in the northwest, central-western, and southeastern counties, while ehrlichiosis is more common in the southern counties. These findings hold true across humans, canines, and deer.

## 1. Introduction

### 1.1. Tick-Borne Diseases: Pathogens and Hosts

The field of disease ecology faces the challenge of linking ecological and epidemiological approaches to better inform human disease risk from vector-borne diseases [1]. While vector-borne diseases are attributable to bacteria, viruses, and parasites transmitted through the bites of ticks, mosquitoes, and fleas [2], the majority of vector-borne diseases in the United States (U.S.) are spread by ticks; these Tick-Borne Diseases (TBDs) accounted for 77% of the 642,602 cases reported to the Centers for Disease Control and Prevention (CDC) between 2004 and 2016, consisting of 16 diseases, including Lyme Disease (LD), Ehrlichiosis (EHR), and Spotted Fever Rickettsioses (SFR) [2]. The CDC notes that these numbers reflect significant under-reporting, such that the true extent of LD and other TBDs is largely undetermined and potentially underestimated [3,4]. *Ixodes scapularis (*I. scapularis)** ticks are known to be widespread in the eastern U.S. However, limited surveillance may account for underreporting outside of the Northeast [5,6,7]; yet the geographic range of *I. scapularis* is spreading rapidly, contributing to the increase in LD cases [6].

Passive tick surveillance is the primary framework for current public health system and practice in the U.S., and relies on reporting of ticks found on people, livestock, or pets, and identification of concomitant disease. Active tick surveillance entails direct collection of ticks from host animals, such as deer and other mammals. Both approaches are limited by time involved in tick collection and testing, and could be improved with more detailed documentation, identification of relevant tick habitats, and incorporation of proxy data associated with TBD risk.

Vector-borne pathogen transmission is affected by a variety of factors from tick ecology, the number of pathogens carried, their reservoirs, and hosts. White-tailed deer (*Odocoileus virginianus*) serve as primary reservoir hosts for Ehrlichia chaffeensis (*E. chaffeensis)*, which is the pathogen that causes human monocytic ehrlichiosis (HME) [8]. The cycle of tick transmission involves feeding on infected reservoir hosts and then transmitting *Borrelia burgdorferi* (Bb) to the host [9]. White-tailed deer (also referred to in this article simply as “deer”) present differing roles as non-competent hosts for LD, but primary hosts for the adult ticks that carry Lyme disease [10]. Unlike small mammals, deer are not reservoirs for LD in particular, but rather are inadvertent hosts. Deer are also noted as important hosts for *E. ewingii,* another pathogen found in humans and canines, transmitted by the Lonestar tick, as well as the Blacklegged Tick *Ixodes scapularis* [11]. The role of wildlife in the circulation of Anaplasma phagocytophilum has yet to be clearly determined, but several species of wild ruminants, including deer, have been considered by some researchers as possibly important reservoirs [12,13]. A natural opportunity for assessing deer density and disease risk occurred on a small island in Denmark, where deer density plummeted following an epidemic; human Lyme neuroborreliosis cases declined in tandem with the host and tick reduction [14]. Despite their resilience to contracting LD, deer nonetheless serve as large “mobile hosts” for pathogen-carrying ticks such as *I. scapularis* infected with bb or other bacterial agents [15]. 

*Ixodes scapularis* ticks responsible for transmitting LD have a broad range of potential hosts—including lizards, rodents, and deer—that may cross paths and transmit infected ticks to humans and/or their pets. The complex feeding cycle of ticks often involves multiple feeding and egg-laying rounds on their deer or rodent hosts before transmission of pathogenic bacteria to a susceptible host; these cycles are impacted by factors such as vegetation, ecological suitability, and a variety of environmental considerations.

However, to date, extant research has not provided definitive resolutions for determining ecological factors associated with human TBD risk. Studies combining ecological, entomological, zoological, and human epidemiological data are scarce. However, importantly, “more targeted tick and pathogen surveillance coupled with studies of human and tick behavior could improve understanding of key risk factors and inform public health interventions.” [1]. 

Noting the gaps in the literature, we introduce a promising approach for determining and informing efforts for monitoring human TBD risk, adopting a broad application of the One Health model, which is interdisciplinary and focuses on the interconnections between humans and animals. Within the One Health Model approach, we draw upon recent research that incorporates triangulation of various fauna-related disease risk proxies to inform public health practitioners and officials about human disease risk by ecosystem and different spatial levels of analysis [16,17,18]. These efforts are of particular importance and used in areas where medical practitioners may be unfamiliar with related disease presentations or lack access to adequate disease surveillance (e.g., outside of the northeastern U.S. region). In states such as Indiana, education levels and access to care vary widely across rural and metropolitan areas. 

This research was guided by a basic question: Does deer population density align with official disease data at the county level? Principal data included (1) human cases of ehrlichiosis, anaplasmosis, Lyme disease, and Spotted Fever Rickettsioses; (2) canine serological reports on ehrlichiosis, anaplasmosis, and Lyme disease; and (3) tick infectivity incidence and levels. We explored these data and related issues using both descriptive statistics to provide estimated associations and means, and mapping to enable visualization and interpretation of spatial trends.

### 1.2. Assessment of Human TBD Risk

Next to mosquitoes, ticks are the most common parasitic disease vector in the world and, in the U.S., ticks are the most prevalent vector for infectious disease, responsible for LD, Rocky Mountain spotted fever and Spotted Fever Rickettsiosis (SFR), Anaplasmosis (ANA), southern tick associated rash illness, and a number of other serious diseases. Active TBD diagnosis, monitoring, and surveillance remain limited by data gaps and time, geography, equipment, funding, and testing opportunities. Carbon dioxide (CO_2_) trapping, removal of ticks from hosts such as deer, birds, lizards, or small mammals, and dragging surveys to collect ticks from their natural environments are some of the most common collection, monitoring, and active surveillance strategies. Although repeated visits and collection provide greater precision in estimating tick density and identifying disease, the limitations are many, including personal risk, securing permits, or identifying suitable habitats. Moreover, these techniques are designed to acquire in situ ticks, and so may be less representative of the tick populations and habitats where most humans are infected with TBDs. Active tick surveillance outcomes also are affected by the sample techniques, such as CO_2_ trapping, rodent collection, or dragging [19]. In a study of ticks in Southern Indiana, tick presence and species were affected by sampling methods as well as climate [19]. To date, and with over 3100 counties and municipalities in the U.S., the information sourced from both active and passive surveillance does not fully inform public health disease prevention, estimation, or intervention.

Passive surveillance that extends beyond simple reporting to public health agencies has the potential to provide further evidence-based, timely, and geographically salient information to medical practitioners, the public, patients, and public health officials. Specifically, the promising use of data proxies, based on, for example, canine, livestock, or other wildlife data can help inform human disease risk at the local level [16,17,18].

### 1.3. Deer Density and Tick-Borne Disease Risk

Deer have been evaluated for their role in spreading tick-borne pathogens, including LD. Deer hosts are not susceptible to Bb infection and, therefore, may dilute the prevalence of these related pathogens and thus reduce infectivity of ticks that feed on them [20]. Conversely, high deer density has the capacity to contribute to disease risk by sustaining a high nymphal tick population [20]. Deer are known to serve as a solid host for blood feeding, even if not suitable reservoirs for Bb [21,22].

Higher densities of *I. scapularis* are found in more forested areas [23], which is where higher levels of deer population density occur and, thus, likely have a higher likelihood of serving as hosts. Deer populations increase the spatial distribution of ticks given that deer habitats serve as primary breeding grounds for wintering tick eggs, even if the deer hosts do not directly spread disease [24]. Adult ticks tend to be more common on deer overall; moreover, adults ticks are less-successful vectors for transmitting LD, as they are more easily detected and removed by their human hosts [24]. Although deer may play a role in the tick life cycle, studies point to a limited association of deer populations with human TBD risk. Some research shows a an increase in LD rates that occurred decades after increases in deer populations, or indicate an association between deer density and increased ticks [25,26,27,28]. Nymphal tick populations were also found to be unaffected by increases in white-tailed deer populations [29,30,31]. Recent research expands active surveillance by assessing deer population movement by sex, differing times of day, and by seasons, finding that deer have expansive reach into residential areas and backyards [32]. Overabundance of deer is also found in areas of low LD risk [33,34].

To date, considerable research on TBD risk is focused on Lyme disease. Scholarly efforts to identify differences in TBDs and human disease risk associated with white-tailed deer by local geographic areas are rare. With the overall purpose of ascertaining the prevalence of deer populations relative to human TBD risk, we used Indiana as a case study due to its detailed and transparent public health reporting, varied ecosystems, and established and closely-monitored deer populations. Additionally, from a public health perspective, Indiana populations vary, substantially in some counties, by educational attainment and health indicators. Our work is informed by and builds upon research incorporating statistical and thematic mapping of official public health-data using human and canine reports of tick-borne diseases by county in Indiana [35]. Based on county-level data, significant spatial variation has been found among ticks in studies conducted in Indiana, showing that their range has been expanding into the Midwest over time, with an increasing geographic prevalence particularly of *I. scapularis* in the region [23].

Related findings are compared to tick presence, including nymphal ticks, and deer density proxies, by county in Indiana. Hunters, as reporters of “deer kills,” have served as observers and checks on the deer population, assisting in building One Health multimodal datasets for determining human disease risk.

### 1.4. Indiana Ecosystems and Deer

Medically important ticks are found throughout Indiana. For example, the American Dog tick is present in all 92 counties, and is known to feed on a wide range of mammals, including deer [36]. The Lonestar tick is found in moist woodlands throughout the state, but primarily in the southern counties. *I. scapularis* is found throughout the state, with adults feeding on deer and other mammals, and nymphal ticks acting as the primary vector of LD transmission to human and canine hosts. The Brown Dog tick, although not native to Indiana, is present in the state and is a known vector of canine EHR [36]. *I. scapularis* is an increasing public health concern across the United States, as it is associated with multiple pathogens such as Lyme disease, anaplasmosis, babesiosis, *Borrelia miyamotoi* disease, Powassan virus disease, and ehrlichiosis, with LD accounting for more than 70 percent of TBD cases [26]. Changes in Indiana’s ecosystems reveal expanding tick migration patterns and disease risks due in large part to climate shifts and environmental changes. For example, work by Keith Clay and his associates involving mapping tick boundaries and disease risk in Indiana has shown that “just in the past 10 years, we are seeing things shift considerably”. As Clay notes, “you used to never see lone star ticks in Indiana; now they are very common.” [15].

**Figure 1:**
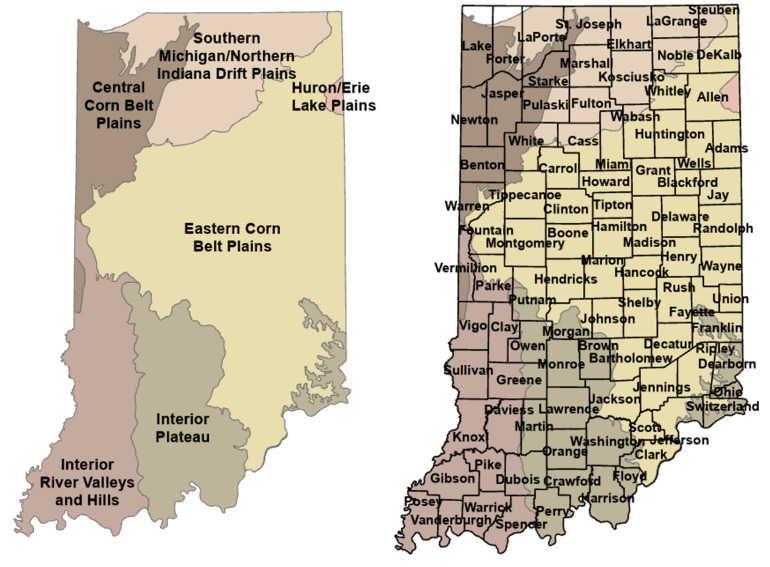
Indiana Ecoregions by County.

Ecoregions are defined by spatial characteristics ranging from hydrology, geology, wildlife, vegetation, and climate (Figure 1). Indiana has eight identified ecoregions containing diverse mixtures of prairies, marshes, dunes, forests, and streams. Within Indiana’s ecosystems, smaller climate and vegetation divergences are notable. For example, within the Central Cornbelt Plains, four separate smaller divisions provide a changing landscape from prairie to marshes and swamp forests [37].

## 2. Materials and Methods

Our analyses employ multi-modal databases in an effort to triangulate indicators that may serve as proxies for human disease risk within the state of Indiana (Table 1). County-level data included human TBD reports confirmed by positive serology and reported to the State by medical practitioners; confirmed tick infectivity; deer density as defined by deer mortality and observations; and canine cases of TBDs.

### 2.1. Data Acquisition

Specifically, five general datasets were consolidated for comparative analysis:

1. **Human TBD Case Rates** for Spotted Fever Rickettsiosis (SFR); Ehrlichiosis (EHR); Anaplasmosis (ANA); Ehrlichiosis or Anaplasmosis, indeterminate (EHRANA); and Lyme Disease (LD) as provided by the Indiana Department of Health (IDOH) via a data request.
2. **Canine TBD Case Rates** for EHR; ANA; and LD, obtained from the Companion Animal Parasite Council’s (CAPC’s) online public data dashboard. The CAPC provides canine serological testing data online [38] via IDEXX Laboratories and IDEXX Diagnostics.
3. **Deer Population** as reported by the Indiana Department of Natural Resources’ (IDNR’s) Annual Deer Reports. Two measures were used:
  (1) **Deer Mortality** is the official number of deer reported as killed (“harvested”) by hunters and vehicle collisions via the “CheckIN Game” (CING) system. Hunters are required to report deer harvest per state law. “Damage Permits” are also issued for hunting deer that are causing property or agricultural damage (e.g., eating crops).
  (2) **Deer Observation** is the rate of deer sightings per hour as estimated by the IDNR’s “Archer’s Index”, a systematic wildlife reporting protocol. These data are thus voluntarily reported by hunters, unlike Deer Mortality.
4. **Tick Infectivity** data for the rate of *Borrelia burgdoferi* infection in adult and nymphal ticks, as provided by the IDOH via a data request.
5. **County-Level** environmental, geographical, and population data obtained from official US reports, including the Decennial Census and the US Geological Survey.

### 2.2. Data Aggregation and Standardization

Where possible, data were acquired and aligned by-county by-year. Indiana has 92 counties and the period of interest (POI) (2017–2020) encompasses 4 years, resulting in 368 county years of data (e.g., Adams County, 2017). Data were aggregated across years to create a “one-way” by-county dataset for the purpose of descriptive spatial statistics; aggregation was performed via sum (total across all years) and via measures of central tendency (mean/median across all years, excluding years with missing or n = 0 data). Aggregated data are reported as n (%), mean (1 standard deviation), and median (interquartile range).

Some data were originally reported across-county. For example, Deer Observations were only provided at the “Deer Management Unit” (DMU) level due to the sparsity of the data at the county-level, as reported by the IDNR. A DMU is a contiguous grouping of counties similar in development, hunter density, and other variables—Indiana’s 92 counties have been organized into 9 DMUs by IDNR via collaboration with Purdue University [39]. Deer Observation data were thus excluded from county-level descriptive statistics and aggregation, and analyses involving Deer Observations required aggregation of the other variable to the DMU-level.

Tick Infectivity data were originally reported by-county but across-year—i.e., the data were already aggregated (summed) across the period of interest (2017 through 2020). We used the aggregated sums as they were in keeping with intentions to aggregate data across years; since we did not have by-year data, we did not calculate by-county means or medians for Tick Infectivity as we did for the other data.

Human and CAPC TBD case data were standardized at the county level as rate per 100,000 people per year using by-county US Decennial Census data. Data standardized using the 2020 US Decennial Census. Other variables—notably Deer Mortality—were not standardized to county populations. This was done because, unlike TBD rates amongst humans or canine pets, deer population is more dependent on natural factors other than human population; e.g., county area, forest cover, or ecoregion. In addition, Deer Observation and Tick Infectivity data were already standardized: the former was provided as a standard “rate per hour”, and Tick Infectivity was standardized as a “percentage of ticks positive for Bb”.

### 2.3. Statistical Analysis

Associations between variables of interest were initially explored using Pearson correlations and simple linear regressions; final estimates were generated using multi-level Mixed Effects Models (MEMs) to account for repeated measures and elapsed time. The generic MEM included the two variables of interest (one considered dependent, the other independent), a continuous variable representing year (to detect any linear year-to-year changes in the dependent variable), and an interaction between the independent variable and year (to detect any changes in the primary association between the independent and dependent variables over time). In addition to these fixed effects, the MEM included random nested effects for County nested within Deer Management Unit (DMU) to account for repeated measures, and a random crossed effect of year to account for year-specific variability separate from any continuous across-year effects.

Every MEM was generated by starting with the base generic model described above. If, after fitting, any of the random effects were observed to have had a negligible effect on the model (i.e., accounted for ~<0.1% of residual variance), they were removed to prevent over-fitting and the MEM re-run. Finally, a Likelihood-Ratio test was conducted for each MEM to determine if it accounted for significant various relative to a simple linear regression. Individual effects were considered statistically significant below a *p*-value of 0.1. Data were organized and groomed in Microsoft Excel for Mac (v16.68), and all analyses were conducted using Stata (v16.1).

### 2.4. Mapping and Visualization

Thematic maps of the deer density proxies and human and canine TBDs by DMU and county in Indiana were created using Tableau 2022.2. In order to determine the best placement of values into different classes in the maps, we used natural breaks classification, the most broadly used method in thematic mapping. This clustering method minimizes the variance within classes while maximizing the variance between classes to find the best breakpoints. We employed ArcGIS Pro 3.0 to map Indiana’s ecoregions because it is more suitable for visualizing geographical features. For this mapping, this study utilized Indiana Level III shape and layer data provided by the U.S. Environmental Protection Agency (EPA).

## 3. Results 

Out of all human TBD cases (n = 1070), just over half (53.9%) were attributed to LD, and approximately a quarter (24.9%) were attributed to SFR. The remainder were predominantly EHR or EHRANA; only 5 ANA cases were reported (Table 2). 

A given county reported an average of 13.7 (19.0) human TBD cases. However, the distribution of human TBD cases was heavily skewed by several high-case counties (such as St. Joseph), with a statistical skewness of 2.58. This can be seen in the noticeably lower median number of human TBD cases reported by a given county of 6.5 (IQR = 15).

When considering counties that reported at least one human case of a given TBD, the TBD with the highest mean cases per county was LD at 8.1 (16.7); after adjusting for population, SFR was observed to have the highest mean rate per county at 6.1 (6.9) cases per 100,000 people. The median cases and rates were more clustered across TBDs, likely due to their skewed distribution across counties: the lowest median rate was observed in EHRANA at 3.5 (4.5), and the highest was EHR at 4.7 (4.6).

Of Indiana’s 92 counties, 77 (83.70%) reported canine serological testing data to the CAPC between the years of 2017 and 2020 (Table 3). Notably, five of these counties that provided data reported conducting no serological tests, leaving 72 (78.26%) counties with at least one reported Canine TBD. The county with the highest number of serological tests conducted was Lake, with 89120 tests conducted throughout the POI. Lake County also reported the highest total number of TBD+ tests; out of 89120 tests conducted, 3078 were LD+ (3.45%), 851 were EHR+ (0.95%), and 370 were ANA+ (0.42%).

Out of all Canine TBD+ cases across all counties (n = 37,787), the large majority were attributed to either LD (60.3%) or EHR (32.8%), with the remaining 6.9% attributable to ANA (Table 3). When considering counties that reported at least one Canine case of a given TBD, the TBD with the highest mean cases per county was LD at 330.2 (583.4); after adjusting for population, LD continued to have the highest rate per county at 137.6 (194.9) cases per 100,000 people. The median cases and rates were more clustered across TBDs, likely due to their skewed distribution across counties: the lowest median rate was observed in ANA at 8.8 (13.6), and the highest was LD at 58.2 (148.7).

All counties in the state of Indiana are required to report various deer mortality metrics to the IDNR. Out of all deer killed (n = 530,002), the vast majority were attributed to hunting permits (87.5%), with vehicle collisions (11.4%) making up most of the remainder; only 1.1% of deer mortality was attributed to damage permits (Table 4). The county with the highest number of deer killed was Steuben, with 12,350 throughout the POI. Steuben County also reported the third highest number of vehicle collisions with deer (n = 1806), following behind Allen (n = 1825) and Kosciusko (n = 1831) Counties. While all counties reported at least one deer death due to hunting and vehicle collisions, 17 (18.5%) counties reported that no deer were killed under damage permits; the highest number of damage permit kills was reported in Washington County (n = 454). 

Tick infectivity data were reported for ticks gathered from 74 (80.4%) of Indiana’s counties, 59 (64.1%) of which reported at least one Bb+ test (Table 5). Out of a total of 4834 ticks tested across all counties, just over a quarter (26.6%) were Bb+. The Bb+ rate was higher in adult ticks (~34.1%) than it was amongst nymphal ticks (~12.9%) across all counties. This trend continued in the by-county Bb+ rates aggregated as means and medians, with adults having a higher Bb+ rate.

### Spatial Mapping and Association

**Figure 2:**
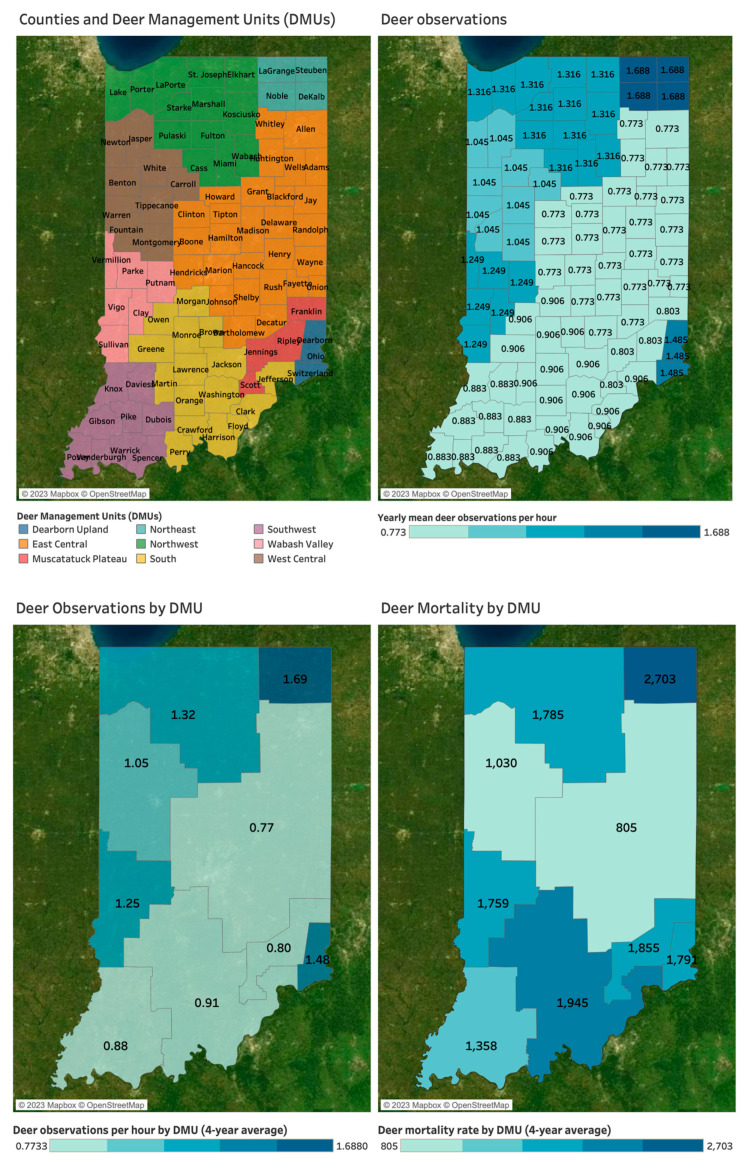
DMUs, deer observations per hour, and deer mortality rate by DMU (4-year average between 2017 and 2020).

Deer in Indiana are abundant and serve as both non-competent hosts and reservoirs for the disease. This study explores the importance of disaggregating disease reports at the county level to distinguish nuances in spatial overlaps among varying types of proxies. The left map in Figure 2 shows nine Deer Management Units (DMUs), contiguous groupings of counties sharing similarities in human development, hunter density, and other variables. The other maps in Figure 2 present deer observation per hour (left map) and deer mortality rate (right map) by DMU level. All values in these two maps indicate a four-year average between 2017 and 2020. The DMU with the highest deer observations per hour is Northeast, followed by Dearborn Upland, Northwest, and Wabash Valley. Similarly, these four DMUs also have relatively high deer mortality rates. However, in the South and Muscatatuck Plateau DMUs, deer observations per hour are low, while the deer mortality rates are high.

Statistically, including a random effect for DMU to account for repeated measures revealed a significant, positive association between these variables (coef. = 2575 Deer Mortality per 1 Deer Observation per Hour, *p* = 0.060).

**Figure 3:**
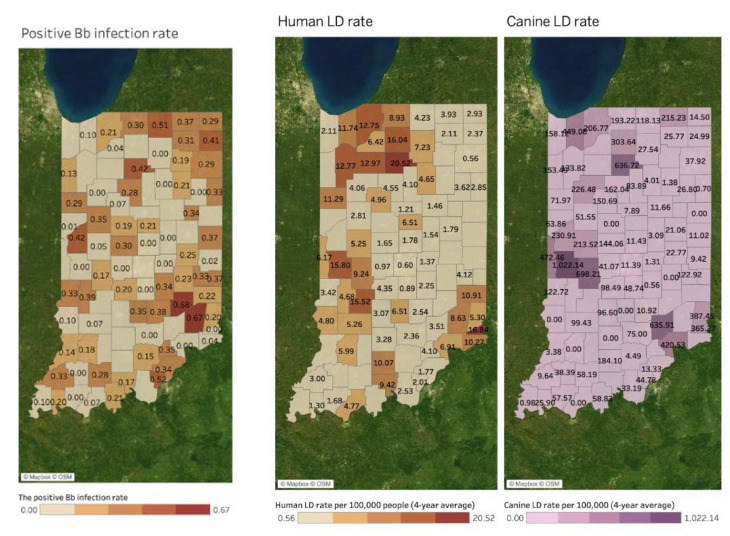
Tick infectivity rate, human LD, and canine LD rates by county (4-year average between 2017 and 2020).

Figure 3 shows spatial patterns of the Bb+ (tick infectivity) rate (left map), human LD rate (middle map), and canine LD rate (right map) by county. Human and canine LD rates indicate the four-year average of LD cases per 100,000 people between 2017 and 2020. Considering most of the canine LD cases are associated with pet owners, it is also standardized by human population size to be directly comparable with human LD rates. A county without a value in the maps indicates a county where disease reports are absent. No value does not necessarily indicate no disease. A similar pattern of hotspots with high rates of LD is found between human and canine LD maps, including northwestern, central western, and southeastern areas in Indiana. The positive Bb infection rate is relatively high in northeastern, central western, and southeastern areas. However, it is not easy to identify apparent hotspots compared to LD maps.

When estimated using the MEM, the association between Human and Canine LD became n.s. (*p* = 0.608); in addition, the effect of time on Human LD rate was n.s. (*p* = 0.320), as was the interaction between time and Canine LD rate (*p* = 0.611). In addition, MEM estimates showed a significant positive effect between Bb+ and Canine LD (2.66 Canine LD cases per 100 k for every 1% higher Bb+, *p* = 0.028) and a weaker positive effect between Bb+ and Human LD that approached significance (0.04 Human LD cases per 100 k for every 1% higher Bb+, *p* = 0.102). Both the Human and Canine LD models showed a strong positive effect of time on case rate, estimating an additional 0.72 Human LD (*p* = 0.001) and 34.02 Canine LD (*p* < 0.001) cases per 100 k people per year.

**Figure 4:**
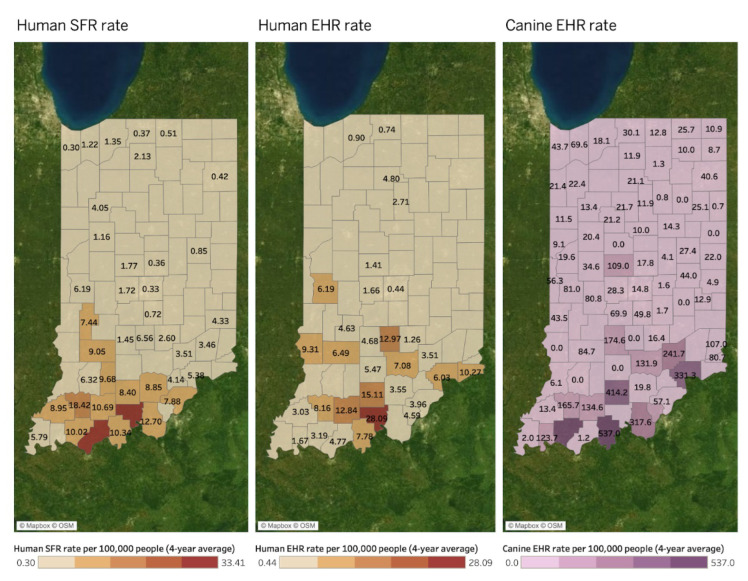
Human SFR and EHR, and canine EHR rates by county (4-year average between 2017 and 2020).

Figure 4 presents similar spatial patterns of human SFR (left map), human EHR (middle map), and canine EHR (right map) rates per 100,000 people. All values in the three maps indicate a four-year average between 2017 and 2020. A county without a value in both maps indicates a county where disease reports are absent. No value does not necessarily indicate no disease. There are spatial similarities among the three TBD rates in southern counties in Indiana. These patterns differ from the positive Bb infection rate and human and canine LD rate in Figure 3 above, which have hotspots in northern and central western counties besides the southern area.

MEM estimates revealed that Canine EHR was negatively associated with Human EHR (−10.11 Human EHR per 1 additional Canine EHR, *p* < 0.001) and positively associated with Human SFR (6.32 Human SFR per 1 additional Canine EHR, *p* = 0.033). There was no significant effect of time on Human EHR (*p* = 0.181) or Human SFR (*p* = 0.362), but significant interactions between time and Canine EHR were observed in both models: the positive association between Canine EHR and Human SFR decreased by −0.003 Human SFR cases per Canine EHR case per year (*p* = 0.033), and the negative association between Canine EHR and Human EHR increased by 0.005 Human EHR cases per Canine EHR per year (*p* < 0.001). A significant, negative association was observed between Human SFR and Human EHR (−616.04 Human EHR per 1 additional Human SFR, *p* < 0.001). Time had a significant positive effect on Human SFR (0.38 Human SFR cases per year, *p* = 0.029) and also significantly interacted with the association between Human SFR and Human EHR: every year increases the positive association by 0.31 Human EHR cases per Human SFR case (*p* < 0.001)

**Figure 5:**
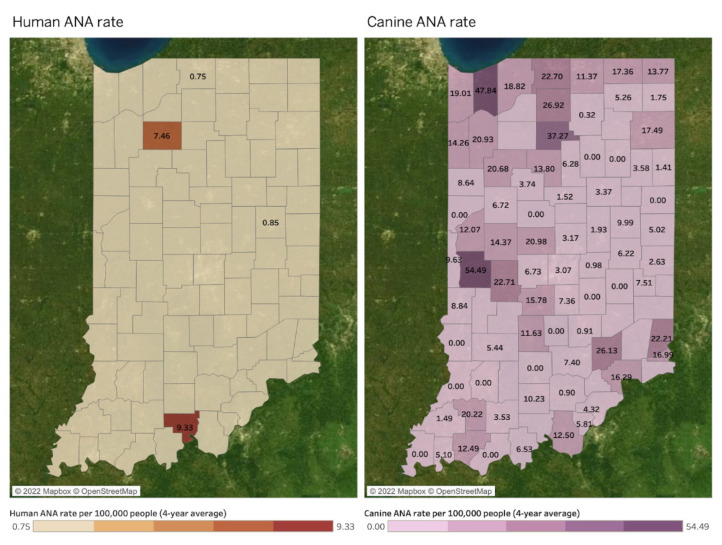
Human and canine ANA rates per 100,000 people by county (4-year average between 2017 and 2020).

Figure 5 illustrates spatial distributions of human ANA (left map) and canine ANA (right map) rates per 100,000 people by county. All values in the three maps indicate a four-year average between 2017 and 2020, and a county without a value in both maps indicates a county where diseases are not reported. The canine LD rate forms hotspots in northwestern and central western counties. However, spatial associations between human and canine ANA rates are not found because of its inefficient data size. 

Statistically, the very small sample—which consists of counties with both Canine and Human ANA (n = 2)—precluded the calculation of regression/correlation coefficients. A Likelihood-Ratio Test of the fitted MEM was n.s. (*p* = 0.320), which indicates that the inclusion of random effects did not improve the model relative to a simple linear regression.

**Figure 6:**
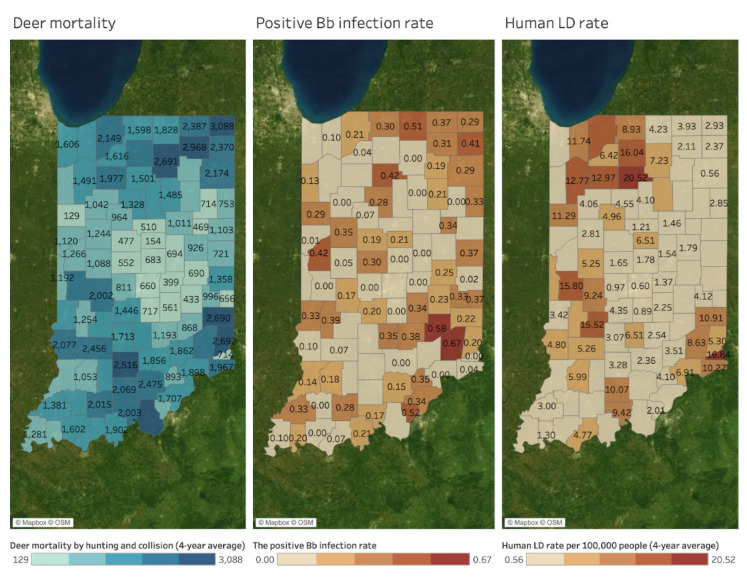
Deer mortality, the positive Bb infection rate, and human LD rate by county (4-year average between 2017 and 2020).

Figure 6 compares spatial patterns of deer mortality by hunting and collision (left map), the positive Bb infection rate (middle map), and the human LD rate per 100,000 people (right map) at the county level. The values in deer mortality and human LD rate maps indicate four-year averages between 2017 and 2020. A county without a value in the maps indicates a county where disease reports are absent, so no value does not necessarily indicate no disease. Deer mortality shares the common hotspots with the positive Bb infection rate in northeastern and southwestern counties. These areas belong to Northeast DMU (northeastern counties) and South, Dearborn Upland, and Muscatatuck Plateau DMUs (southeastern counties). On the other hand, deer mortality shows spatial similarities with human LD rate in the northwestern and central western areas, which shares common part with Northwest DMU (northwestern counties), Wabash Valley DMU (central western counties), and South, Dearborn Upland, and Muscatatuck Plateau DMUs (southeastern counties). However, they are not apparent compared to the positive Bb infection rate.

The MEM-estimated association between Human LD and Deer Mortality was negative and n.s. (−2818 Deer Mortality per additional 1 Human LD case per 100 k, *p* = 0.355). The model included a significant positive time effect on Deer Mortality (31 Deer Mortality per year, *p* = 0.032); the interaction between time and the association between Deer Mortality and Human LD was n.s. (1.40 Deer Mortality per Human LD case per year, *p* = 0.355).

While a very weak positive correlation was observed between Deer Mortality and Bb+ in Pearson’s correlations and simple linear regressions (*p* = 0.9313), theMEM model estimated that their association was significant and positive (6.92 Deer Mortality per additional 1% Bb+, *p* = 0.052). Deer Mortality was also observed to significantly increase over time (34.19 Deer Mortality per year, *p* = 0.014).

**Figure 7:**
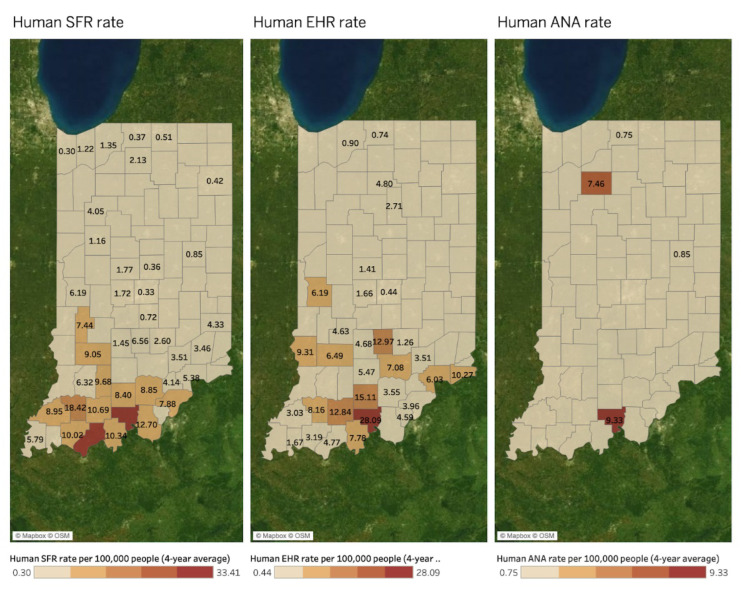
Deer mortality and human SFR, EHR, and ANA rates per 100,000 people by county (4-year average between 2017 and 2020).

Figure 7 shows spatial distributions of human SFR (left map), EHR (middle map), and ANA (right map) rates per 100,000 people at the county level. These are four-year averages between 2017 and 2020. Since a county without a value in the maps indicates a county where disease reports are absent, no value does not necessarily indicate no disease. Unlike the human LD rate, hotspots with high human SFR and EHR rates are in southern Indiana counties. In terms of DMU, Southwest DMU and South DMU cover the hotspots. However, due to the small data size of human ANA rates, it is hard to identify the spatial similarities with deer mortality by hunting and collision.

Statistically, the very small sample size of counties with both Human ANA and Human EHR (n = 2), or with Human ANA and Human SFR (n = 3), precluded the calculation of regression/correlation coefficients. In addition, a Likelihood-Ratio Test of the Human ANA/EHR MEM was n.s. (*p* = 0.320), which indicates that the inclusion of random effects did not improve the model relative to a simple linear regression. However, the MEM for Human SFR and Human ANA had a significant Likelihood-Ratio Test (*p* = 0.0046), and estimated a significant negative association (−99.59 Human ANA per 1 additional Human SFR, *p* < 0.001). While time had a n.s. effect on Human ANA (*p* = 0.650), the negative association between Human ANA and Human SFR appeared to significantly strengthen over time (−0.14 Human ANA cases per 1 additional Human SFR case per year, *p* < 0.001).

**Figure 8:**
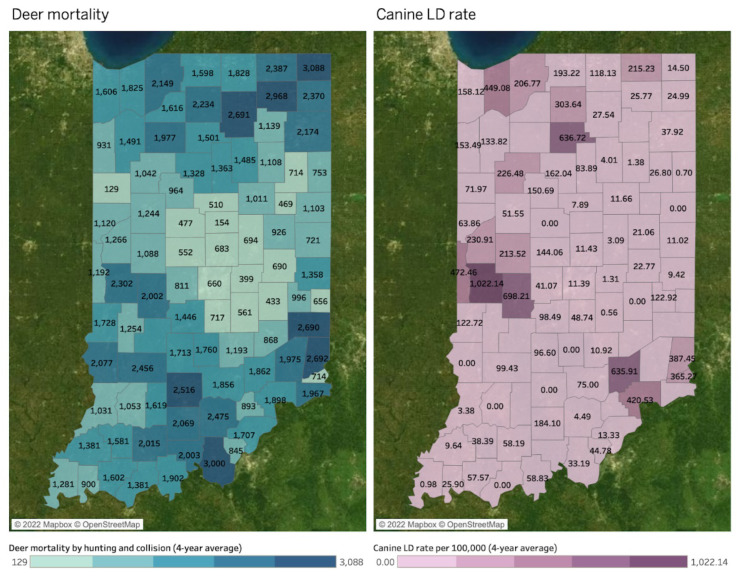
Deer mortality and canine LD rate by county (4-year average between 2017 and 2020).

Figure 8 compares spatial similarities of deer mortality by hunting and collision (left map) and canine LD rate per 100,000 people at the county level. These are four-year averages between 2017 and 2020, and a county without a value in the maps indicates a county where disease reports are absent. Lack of canine data does not imply no disease. The deer mortality map shares the common hotspots with high canine LD rates in northeastern, central western, and southwestern counties, similar to the human LD rate. These counties are covered by Northeast DMU (northeastern counties), Wabash Valley (central western counties), and parts of South, Dearborn Upland, and Muscatatuck Plateau DMUs. 

When modeled via MEM, the association between Canine LD and Deer Mortality was positive but not significant (*p* = 0.398), and this association did not significantly vary over time (*p* = 0.398). However, Deer Mortality significantly increased over time in the model (44.83 Deer Mortality per year, *p* = 0.004).

**Figure 9:**
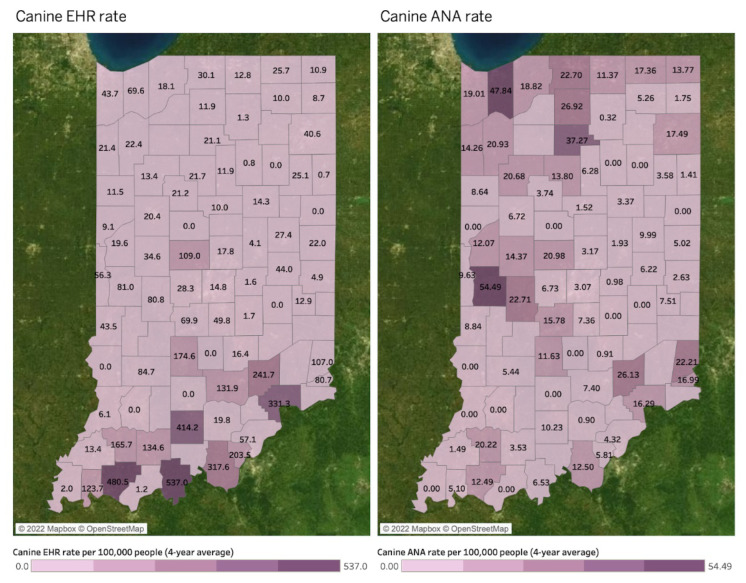
Deer mortality and canine EHR and ANA rates by county (4-year average between 2017 and 2020).

Figure 9 visualizes spatial patterns of canine EHR (left map) and ANA (right map) per 100,000 people at the county-level to compare with deer mortality by hunting and collision. Numbers in the maps are four-year average values between 2017 and 2020, and a county without a value indicates a county where disease reports are absent. Unlike the canine LD rate, the canine EHR rate has a hotspot in the southern area where deer mortality is high. The hotspot belongs to Southwest, South, and Muscatatuck Plateau DMUs. On the other hand, canine ANA shows similar distribution to canine LD rate, which has high rates in northwestern and central western counties. These counties are covered by Northeast DMU (northeastern counties) and Wabash Valley (central western counties). 

A MEM estimated that the association between Canine EHR and Canine ANA was in fact negative, albeit n.s. (*p* = 0.408), and did not change over time (*p* = 0.408). The model estimated a significant increase in Canine EHR over time (12.23 Canine EHR cases per 100 k per year, *p* = 0.011).

Table 6A,B summarize the outcomes of the bivariate associations presented above, as well as several additional exploratory comparisons conducted after the spatial analysis. Deer Mortality was significantly positively associated with both Human EHR (24797 Deer Mortality per Human EHR case per 100 k, *p* = 0.023) and Canine EHR (923 Deer Mortality per Canine EHR case per 100 k, *p* < 0.001). Both models also reported significant increases in Deer Mortality over time (Human EHR: 44.32 Deer Mortality per Year, *p* = 0.006; Canine HER: 71.74 Deer Mortality per Year, *p* < 0.001), and indicated that the positive association between Deer Mortality and EHR is significantly decreasing over time (Human EHR: −12.29 Deer Mortality per Human EHR case per 100 k per Year, *p* = 0.023; Canine EHR: −0.46 Deer Mortality per Canine EHR case per 100 k per Year, *p* < 0.001). Lastly, Tick Infectivity was analyzed for association with EHR and SFR: all associations were weakly negative, with the largest effect observed between Bb+ and Human SFR (−0.05 Human SFR cases per 100 k per 1% Bb+, *p* = 0.020), and the association between Bb+ and Human EHR was similarly significant (−0.04 Human EHR cases per 100 k per 1% Bb+, *p* = 0.004). However, the association between Bb+ and Canine EHR was n.s. (*p* = 0.316). In all three models, TBD case rates were significantly influenced by time: EHR significantly increased over time in both Humans (0.62 Human EHR per 100 k per year, *p* = 0.002) and Canines (18.98 Canine EHR per 100 k per year, *p* < 0.001). In contrast, Human SFR exhibited a significant downward trend over time (−0.54 Canine EHR per 100 k per year, *p* = 0.009).

**Figure 10:**
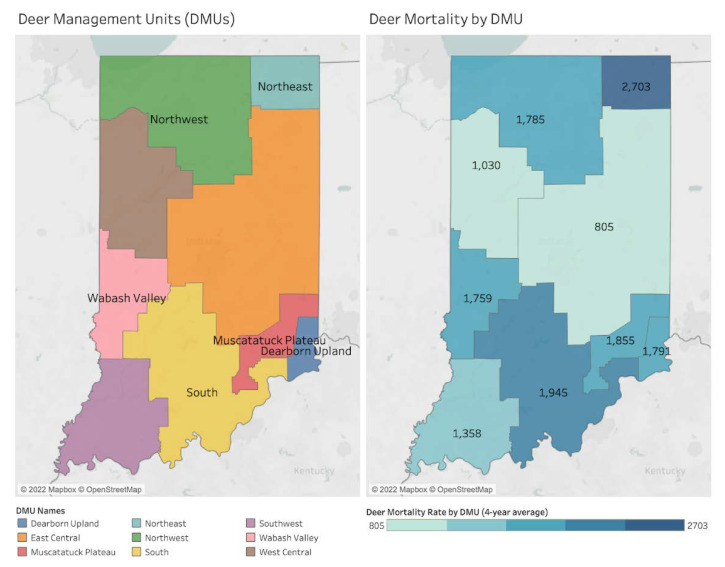
Deer mortality rates by DMU (4-year average, 2017–2020).

Table 7 summarizes hotspots of deer mortality and human and canine TBD rates in Indiana at the DMU-level, as also shown in Figure 10. The first column indicates hotspots by DMU, and the second column shows the average deer mortality rate by DMU. The DMU with the highest average deer mortality rate was Northeast (2703) DMU, followed by South (1945), Muscatatuck Plateau and Dearborn Upland (1855 and 1791), Northwest (1785), and Wabash Valley (1759) DMUs. In TBD-related columns (third to last columns), ‘O’ indicates that each TBD rate shares spatial similarities with deer mortality pattern. For example, the positive Bb infection rate has two ‘O’s, which means its hotspots are distributed in Northeast and Muscatatuck Plateau & Dearborn Upland DMUs. Human and canine LD rates both are high in Muscatatuck Plateau & Dearborn Upland, Northwest, and Wabash Valley DMUs. Human SFR, human EHR, and canine EHR have hotspots in South DMU. Though the canine ANA rate is high in Northeast and Wabash Valley DMUs, the human ANA rate does not show any cluster because of its inefficient reported cases for mapping. These imply that deer population density could be related to human and canine TBD distributions. 

## 4. Discussion

By employing a One Health perspective and triangulating multi-modal animal and human data, we sought to understand and assess human Tick-Borne Disease (TBD) risk. Our work builds upon recent research that incorporates county-level data on TBDs matched with other national and state information from scholarly and official sources to determine risk patterns [19,40]. The One Health Model is supported by the CDC and focuses on the connectivity among animals, humans, and zoonotic disease. The lack of county-level data available to public health practitioners and researchers from official sources such as the CDC on TBDs other than LD has created an immediate need for innovative approaches to determining the factors in pathogen transmission. Simply stated, “Where ticks are found, tick-borne diseases can present a threat to human and animal health” [41]. The World Health Organization (WHO) recommends the adoption of a One Health Model to assess TBD risk among humans and animals worldwide [42]. Hosts and vectors engage in complex environments that are variable in nature, prompting scholars to call on veterinarians and medical practitioners to unite in addressing TBDs [43]. The current paper expanded on One Health Model premises and introduced triangulation beyond canines and humans, extending innovative analyses to include wildlife and tick infectivity. 

Using data drawn from county-level databases on deer mortality, tick infectivity, and TBD rates amongst canines in Indiana, as well as spatial overlap with ecosystem regions, associations between these factors and human TBD cases were analyzed via Mixed Effect Models and mapping to better determine the extent to which deer are a potential means of vector transmission at a granular spatial level, along with associated potential proxies of disease risk. Findings suggest that passive One Health surveillance using mutli-modal databases with specific proxies can provide a robust alternative for indicating human disease risk. 

Using multimodal data analysis using a variety of proxies to better estimate disease risk, we find similar spatial distributions between deer population density and human and canine TBDs in northeastern and southern Indiana, which are rural and mixed geographic areas. Overall, LD is more prevalent in the northwest, central-western, and southeastern counties, while ehrlichiosis is more common in the southern counties. These findings hold true across humans, canines, and deer, which is notable given the varying tick vectors that carry both human and canine ehrlichiosis in particular. 

Previous research has demonstrated that sources other than official public health data—such as canine serological reports or self-reported human tick bite encounters—may offer robust proxies for determining TBD risk among humans [16,17,18,39]. Moreover, current scholarship indicates that additional research is needed to assess deer as accurate proxies for human TBD risk. Deer are known hosts for medically important ticks, making them potential proxies for understanding human disease risk at a larger scale than local ecosystems.

Our mixed effect analysis showed a significant positive association of Deer Mortality with EHR in both canines and humans. This suggests that deer may be a useful proxy for EHR and similar TBDs, such as SFR (which was observed to also have a strong positive association with Deer Mortality). We used two variables to estimate overall deer population: Deer Mortality, the official number of deer fatalities in each county, and Deer Observations, a separate official measure of deer population based on in situ sightings by hunters in each DMU. Unlike Deer Mortality, Deer Observation is reported via a survey, includes fawns in addition to adults, and is standardized into “deer observations per hour”; accordingly, as a scientific measurement, Deer Observations are more flexible and adaptable than gross deer fatalities. Deer Mortality and Deer Observations were significantly positively correlated with each other, supporting the validity of Deer Observations as another measure of deer population. 

LD rates between humans and canines were not significantly associated with each other; ANA also was not significant between species, although this may be attributable to the relative rarity of ANA cases in Indiana. Human EHR and SFR were both significantly associated with Canine EHR. Interestingly, Canine EHR was negatively correlated with Human EHR, yet positively correlated with Human SFR. Indiana’s human TBD reporting also distinguishes between SFR and EHR. However, the CAPC does not test for SFR in Canines, so there is no opportunity for EHR and SFR to be conflated in the Canine data. It may be that the positive association between Canine EHR and Human SFR in Indiana—in addition to the significant negative association between EHR and SFR in Humans—is similar to a situation in Virginia where-by an apparent increase in SFR was actually due to an increase in EHR. Gaines et al. noted that a reported increase in SFR cases in Virginia may in fact be mis-reported EHR and related diseases, due to flaws in how physicians and commercial laboratories test and report outcomes to the state departments, and based on testing of tick vectors [44]. 

Tick Infectivity was positively correlated with LD in canines and humans. This is not particularly unusual, as Tick Infectivity was specifically based on the detection rate of *Borrelia burgdoferi*, the primary bacterial agent that causes LD, while EHR and SFR are caused by a different family of bacteria (*Anaplasmataceae*). However, Tick Infectivity (i.e., the presence of *B. burgdoferi*) was also negatively correlated with the case rates of EHR and SFR in humans (n.s. with EHR in Canines). This suggests that there may be competition between Anaplasmataceae and Borreliacaea over limited tick reservoirs and their associated vectors (e.g., deer). In addition, local spatial factors—e.g., climate or access to forested areas—may influence the infectivity of these bacterial families via interactions with their environment and hosts, resulting in regions more susceptible to LD or to EHR/SFR. Interestingly, Deer Mortality was significantly positively correlated with Tick Infectivity—i.e., the more deer in a county, the more likely ticks in that county hosted *B. burgdoferi*. This observation may be explained by ticks on deer being a more suitable and/or effective host for *B. burgdoferi*, whereas *Anaplasmataceae* bacteria are better suited to ticks on other host animals.

Importantly, studies to date largely have not disaggregated deer population data by both county and disease. Nor do they typically compare TBDs separately across small spatial units of analysis, such as counties, or within ecosystems. Additionally, the importance of smaller mammals in the spread of Borrelia to ticks has been identified, as these fauna serve as competent hosts for the pathogen [27,30,32]. For example, dusky-footed woodrats have been shown elsewhere to be key factors in human disease risk, implying that peri-domestic exposure is more salient than recreational exposure in assessing TBD risk [1].

We also observed relevant patterns that may inform additional studies focusing on the presence of specific ticks—and their animal vectors—relative to geographical and ecological factors, and considering the potential drawbacks of current standards for clinical TBD testing and reporting. Spatial overlap of both Human and Canine LD, as well as Canine ANA, was indicated in northwestern and southeastern areas of Indiana. Similarly, EHR was found in the south-central area of the state among both Canines and Humans. This is an area of higher deer density, and white-tailed deer are known reservoirs of the ticks and bacteria known to cause EHR. Accordingly, health officials might act to raise public awareness of high risks of tick bites in brushy or wooded areas in these counties in particular. 

In sum, public health risks are occurring in the face of continued increases in tick-borne diseases across the United States. However, there is a lack of disease-specific information from active or passive surveillance to effectively distinguish disease risk among humans. Those afflicted find themselves in search of diagnosis and report traveling more than 50 miles for medical care and waiting more than seven years for diagnostic confirmation [40]. This research suggests that county-level data may prove useful, providing greater insights and information for application in this regard. As counties in Indiana vary in population density, age, incomes, education levels, and access to healthcare, different individuals and groups may be at higher risk and can face inequitable diagnosis and treatment without proper disease-risk data. Additionally, environmental factors continue to impact both the explosion of vector-borne diseases in some regions and the uneven distribution of risk and access to healthcare [45]. There remains a lack of highly sensitive and specific direct detection methods for LD, relying mainly on antibody testing. Seronegative LD testing also confounds data issues, as some patients may develop chronic symptoms sans a positive antibody test [46,47]. Case reports also indicate Bb relapses following multiple courses of antibiotic therapy [48]. 

This study has offered a way to pinpoint disease risk in different geographic locations, demonstrating the usefulness of triangulating multimodal databases to generate relevant information. Related findings imply that public health systems may be able to determine risk factors at the local level and develop appropriate prevention materials, including clinical symptoms of diseases such as EHR. While not nearly as notorious as LD, EHR and other TBDs are increasingly prevalent, posing critical personal and public health risks.

### Limitations

This study was exploratory in nature, pointing to areas and opportunities for further research, which also are revealed in the study limitations. First, there was a limited number of years due to data availability, including data on tick infectivity, which was provided pre-aggregated for the years 2017–2020. Future work should capitalize on the tentative results presented here by incorporating TBD data (or potential proxies of such) from other databases, and/or replicating our approach in regions with more available data and robust data collection systems (e.g., other states). Additionally, data regarding other tick-borne diseases, such as babesiosis, bartonellosis and tick-transmissible viral pathogens (e.g., Powassan, Bourbon virus, and other emerging viral pathogens), were not available for this study. Lastly, the skewed distribution of both human and canine TBD data across counties indicates that future work should consider analyzing data at the DMU-level rather than the county-level; while county-level effects are almost certainly present, the physical region of DMUs were defined statistically based on geographic and ecological data, whereas counties were primarily defined by sociopolitical effects presumably independent of deer, tick, and TBD occurrence. In addition, the high number of counties increases the number of observations needed; aggregating by-DMU could identify gross effects at the DMU-level, which could then be explored with targeted county-level analyses.

Tick infectivity is a difficult variable with which to work, as active surveillance and testing of ticks depend on researchers, funding, and geographic location selection. For tick infectivity, a lack of data does not necessarily equate to zero ticks or no disease. Second, canine data accounts for only approximately 30% of all positive canine disease cases [49]. As with active tick collection, lack of canine data does not imply no disease; some counties are rural and may have limited access to veterinarians capable of serological testing and who submit these results to the CAPC. A third limitation is human data, which is reported to the state by clinicians. Many patients will be diagnosed after a clinical examination and/or without testing, and these would not appear in the dataset. In the thematic maps, there are counties where disease reports are absent.

## 5. Conclusions

This study extends public health entomological efforts by including multi-modal datasets to assess human TBD risk. We investigate deer density and other One Health indicators, using both spatial and statistical approaches. The analyses conducted in this study suggest the need for finer-grained, disaggregated data on tick-borne diseases. In general, spatial patterns reveal positive associations among diseases, but the geographic alignments are unique. It is a noteworthy result that the highest frequency of human tick-borne diseases occurred in three contiguous northwest counties. Overall, Lyme disease is more prevalent in the northwest, central-western, and southeastern counties, while ehrlichiosis is more common in the southern counties. These findings hold true across humans, canines, and hunting data, which served as a proxy for deer density.

## Figures and Tables

**Table 1:** Definition of variables and data sources. IDOH = Indiana Department of Health; CAPC = Companion Animal Parasite Council; IDNR = Indiana Department of Natural Resources; US = United States.

Variable Name	Definition (4-Year Average between 2017 and 2020) ^1^	Data Source
Human LD	Human LD rate per 100,000 people	IDOH
Human SFR	Human SFR rate per 100,000 people	IDOH
Human EHR	Human EHR rate per 100,000 people	IDOH
Human ANA	Human ANA rate per 100,000 people	IDOH
Human EHRANA	Human EHRANA rate per 100,000 people	IDOH
Canine LD	Canine LD rate per 100,000 people	CAPC ^2^
Canine EHR	Canine EHR rate per 100,000 people	CAPC ^2^
Canine ANA	Canine ANA rate per 100,000 people	CAPC ^2^
Deer Observation	The number of deer observations per hour	IDNR ^3^
Deer Mortality	The number of deer deaths by hunting and collision	IDNR ^3^
Tick Infectivity	The positive infection rate of *Borrelia burgdorferi*	IDOH
County Population ^4^	2010 and 2020 Decennial US Censuses	US Census ^5^

^1^ For information on Tick Infectivity indicators, please see description in Section 2.2; ^2^ CAPC: https://capcvet.org/maps#/2020/all-year/lyme-disease/dog/united-states (accessed 15 December 2022); ^3^ IDNR: https://www.in.gov/dnr/fish-and-wildlife/wildlife-resources/animals/white-tailed-deer/county-data/ (accessed 15 December 2022); ^4^ County populations as reported by the official US Decennial Censuses were used to convert frequencies into rates (per 100,000 people); ^5^ U.S. Census: https://data.census.gov/ (accessed 15 December 2022).

**Table 2:** Summary statistics of Human TBD frequency across 2017–2020 as reported by the IDOH. In addition to the number (%) of counties with at least one of each TBD case and the total number (%) of cases across counties, the table provides means and medians both for the raw total number of cases and the rate of incidence per 100,000 people. Only TBD+ counties were considered when calculating these statistics. TBD = Tick-borne Disease; IDOH = Indiana Department of Health; EHR = Ehrlichiosis; ANA = Anaplasmosis; LD = Lyme Disease; SD = Standard Deviation; IQR = InterQuartile Range.

	SFR	EHR	ANA	EHRANA	LD	ANY TBD
TBD+ Counties, n (%)	43 (46.7%)	32 (34.8%)	4 (4.3%)	39 (42.4%)	71 (77.2%)	78 (84.8%)
Cases, n (%)	266 (24.9%)	109 (10.2%)	5 (0.5%)	113 (10.6%)	577 (53.9%)	1070 (100.0%)
Cases, Mean (SD)	6.2 (7.5)	3.4 (3.1)	1.3 (0.5)	2.9 (2.6)	8.1 (16.7)	13.7 (19.0)
Cases, Median (IQR)	3.0 (5.0)	2.0 (3.5)	1.0 (0.5)	2.0 (3.0)	3.0 (7.0)	8.0 (16.0)
Rate, Mean (SD)	6.1 (6.9)	6.0 (5.5)	4.6 (4.5)	4.8 (4.8)	5.6 (4.5)	9.2 (8.4)
Rate, Median (IQR)	4.1 (7.5)	4.7 (4.6)	4.2 (7.6)	3.5 (4.5)	4.1 (5.0)	6.3 (10.1)

**Table 3:** Summary statistics of canine TBD frequency across 2017–2020 as reported by the CAPC. In addition to the number (%) of counties with at least one of each TBD case, and the total number (%) of cases across counties, the table provides means and medians both for the raw total number of cases and the rate of incidence per 100,000 people. Only TBD+ counties were considered when calculating these statistics. TBD = Tick-borne Disease; CAPC = Companion Animal Parasite Council; EHR = Ehrlichiosis; ANA = Anaplasmosis; LD = Lyme Disease; SD = Standard Deviation; IQR = InterQuartile Range.

Canine TBD	Tested	LD+	EHR+	ANA+	ANY TBD+
Counties, n (%)	72 (78.3%)	69 (75.0%)	69 (75.0%)	63 (68.5%)	70 (76.1%)
Total Cases, n (%)	634,586 (100.0%)	22,782 (3.6%)	12,400 (2.0%)	2605 (0.4%)	37,787 (6.0%)
Cases per County, Mean (SD)	8813.7 (14481.7)	330.2 (583.4)	179.7 (261.1)	41.4 (72.8)	539.8 (784.8)
Cases per County, Median (IQR)	3608.0 (9716.0)	128.0 (347.0)	53.0 (215.0)	20.0 (38.0)	284.5 (655.0)
Rate per 100 k per County, Mean (SD)	2508.7 (2239.2)	137.6 (194.9)	70.7 (112.0)	11.9 (10.7)	216.0 (245.8)
Rate per 100 k per County, Median (IQR)	2307.1 (2738.3)	58.2 (148.7)	22.1 (68.8)	8.8 (13.6)	149.0 (230.1)

**Table 4:** Summary statistics of deer mortality across 2017–2020 as reported by the IDNR. Deer mortality can be attributed to three main causes: killed under a hunting permit (“harvested”), killed by a vehicle collision, or killed under a special damage permit issued to farmers for deer who cause agricultural/property damage. The table presents the number (%) of counties with at least one instance of each type of mortality, and the total number (%) of kills across counties as both means and medians. Only counties with at least one instance of mortality were considered when calculating these statistics. Deer Mortality was not standardized to county population, so no Rates per 100 k are reported. IDNR = Indiana Department of Natural Resources; SD = Standard Deviation; IQR = InterQuartile Range.

Deer Mortality	All Mortality	Harvest	Collisions	Damage Permits
Counties, n (%)	92 (100.0%)	92 (100.0%)	92 (100.0%)	75 (81.5%)
Total Mortality, n (%)	530,002 (100.0%)	463,700 (87.5%)	60,269 (11.4%)	6033 (1.1%)
Mortality by County, Mean (SD)	5760.9 (2760.0)	5040.2 (2512.5)	655.1 (387.7)	80.4 (104.5)
Mortality by County, Median (IQR)	5487.0 (4079.8)	4709.5 (3949.8)	582.5 (406.3)	42.0 (84.0)

**Table 5:** Summary statistics of tick infectivity across 2017–2020 as reported by the Indiana Department of Health. Ticks—divided into Adults and Nymphs—were submitted for testing were assayed for the presence of Bb, the main bacterial agent responsible for Lyme Disease. The table presents the number (%) of counties that tested ticks or at reported least one Bb+ tick (see column headers), and the total number (%) of tested ticks or Bb+ ticks across counties as means and medians, both for the raw totals and the percentage rate of Bb+ infectivity. Only counties with at least one tested tick or Bb+ tick (see column headers) were considered when calculating these statistics. IDOH = Indiana Department of Health; Bb = *Borrelia burgdorferi*; SD = Standard Deviation; IQR = InterQuartile Range.

Tick Infectivity	Tested, All	Bb+, All	Tested, Adult	Bb+, Adult	Tested, Nymph	Bb+, Nymph
Counties, n (%)	74 (80.4%)	59 (64.1%)	72 (78.3%)	57 (62.0%)	62 (67.4%)	38 (41.3%)
Total Ticks, n (%)	4834 (100.0%)	1288 (26.6%)	3139 (64.9%)	1070 (22.1%)	1695 (35.1%)	218 (4.5%)
Ticks per County, Mean (SD)	65.3 (65.9)	21.8 (24.7)	43.6 (44.4)	18.8 (21.3)	27.3 (26.8)	5.7 (5.0)
Ticks per County, Median (IQR)	52.0 (72.0)	15.0 (30.0)	33.0 (45.0)	13.0 (21.0)	20.0 (34.0)	4.0 (6.0)
Bb+ Rate, Mean (SD)	--	25.9% (14.1%)	--	33.6% (18.7%)	--	15.9% (9.0%)
Bb+ Rate, Median (IQR)	--	27.6% (18.2%)	--	36.2% (26.3%)	--	14.2% (10.6%)

**Table 6:** (**A**) Summary of Mixed Effects Models (MEMs) conducted between variable pairs. Each pair lists the model components and parameters used in their final MEM: the level of analysis (DMU or County) and the inclusion of the fixed effect of Year (i.e., time), of the fixed effect of Year’s interaction with the Main Effect Each, the random nested effect of DMU, the random nested effect of County, and the random crossed-effect of Year across the nested DMU > County groups. Each pair includes Table 6 of its associated spatial comparison map, if applicable. DMU = Deer Management Unit; MEM = Mixed Effects Model; LD = Lyme Disease; Bb = *Borrelia burgdorferi*; Bb+ = Percentage of ticks infected with Bb; EHR = Ehrlichiosis; SFR = Spotted Fever Rickettsiosis; ANA = Anaplasmosis. (**B**) Summary of the outcomes of the Mixed Effects Models (MEMs) conducted between variable pairs. Each pair lists the Main Effect (i.e., of the Independent Variable on the Dependent Variable), Time Effect (i.e., of Year on the Dependent Variable), and Interaction Effect (i.e., of Year on the Main Effect); all effects are listed in format Coefficient (Standard Error), with their associated significance indicated by ** (*p* < 0.05), * (*p* < 0.1), or no asterisk (*p* ≥ 0.1). Effects that could not be estimated are indicated by a double dash (--). Each pair also lists its models’ number of observations (n), the total number of nested groups (k), and the outcome of a Likelihood-Ratio test conducted between the model and a simple linear regression. Each pair includes Table 6 of its associated spatial comparison map, if applicable. DMU = Deer Management Unit; MEM = Mixed Effects Model; LD = Lyme Disease; Bb = *Borrelia burgdorferi*; Bb+ = Percentage of ticks infected with Bb; EHR = Ehrlichiosis; SFR = Spotted Fever Rickettsiosis; ANA = Anaplasmosis.

**(A)**								
**Figure Number**	**Independen*t* Variable**	**Dependent Variable**	**County- or DMU-Level**	**Year (Fixed Covariate)**	**Year (Fixed Interaction with Ind. Var.)**	**Year (Random Crossed Effect)**	**DMU (Random Nested Effect)**	**County (Random Nested Effect)**
Figure 2	Deer Mortality	Deer Observations	DMU	No	No	No	Yes	N/A
Figure 3	Bb+	Canine LD	County	Yes	No	No	Yes	Yes
Figure 3	Canine LD	Human LD	County	Yes	Yes	No	Yes	Yes
Figure 3	Bb+	Human LD	County	Yes	No	Yes	Yes	Yes
Figure 4	Canine EHR	Human EHR	County	Yes	Yes	Yes	Yes	Yes
Figure 4	Human SFR	Human EHR	County	Yes	Yes	Yes	Yes	No
Figure 4	Canine EHR	Human SFR	County	Yes	Yes	No	Yes	Yes
Figure 5	Canine ANA	Human ANA	County	Yes	Yes	Yes	No	No
Figure 6	Human LD	Deer Mortality	County	Yes	Yes	Yes	Yes	Yes
Figure 6	Bb+	Deer Mortality	County	Yes	No	Yes	Yes	Yes
Figure 7	Human EHR	Human ANA	County	Yes	Yes	No	Yes	No
Figure 7	Human SFR	Human ANA	County	Yes	Yes	No	Yes	No
Figure 8	Canine LD	Deer Mortality	County	Yes	Yes	Yes	Yes	Yes
Figure 9	Canine ANA	Canine EHR	County	Yes	Yes	Yes	Yes	Yes
Figure 8 and Figure 9	Canine EHR	Deer Mortality	County	Yes	Yes	Yes	Yes	Yes
Figure 6 and Figure 7	Human EHR	Deer Mortality	County	Yes	Yes	Yes	Yes	Yes
Figure 3 and Figure 4	Bb+	Canine EHR	County	Yes	No	No	Yes	Yes
Figure 3 and Figure 4	Bb+	Human EHR	County	Yes	No	Yes	Yes	Yes
Figure 3 and Figure 4	Bb+	Human SFR	County	Yes	No	No	Yes	Yes
**(B)**								
**Figure Number**	**Independen *t* Variable**	**Dependent Variable**	**Observations (n)**	**Total Nested Groups (k)**	**Main Effect (Ind. Var. × Dep. Var.)**	**Time Effect (Year × Dep. Var.)**	**Interaction (Time × Main Effect)**	**LR-Test *p*-value**
Figure 2	Deer Mortality	Deer Observations	36	9	2575.08 (1368.5) *	--	--	<0.001
Figure 3	Bb+	Canine LD	280	77	2.66 (1.21) **	34.02 (6.34) **	--	<0.001
Figure 3	Canine LD	Human LD	280	77	0.84 (1.64)	0.18 (0.18)	0 (0)	<0.001
Figure 3	Bb+	Human LD	368	92	0.04 (0.02)	0.72 (0.22) **	--	<0.001
Figure 4	Canine EHR	Human EHR	280	77	−10.11 (1.73) **	0.17 (0.13)	0.01 (0) **	0.001
Figure 4	Human SFR	Human EHR	368	9	−616.04 (47.25) **	0.38 (0.17) **	0.31 (0.02) **	0.005
Figure 4	Canine EHR	Human SFR	280	77	6.32 (2.97) **	−0.16 (0.18)	0 (0) **	<0.001
Figure 5	Canine ANA	Human ANA	280	1	--	--	--	0.336
Figure 6	Human LD	Deer Mortality	368	92	−2817.75 (3043.74)	31.16 (14.56) **	1.4 (1.51)	<0.001
Figure 6	Bb+	Deer Mortality	368	92	6.92 (3.55) *	34.19 (13.88) **	--	<0.001
Figure 7	Human EHR	Human ANA	368	9	--	--	--	0.320
Figure 7	Human SFR	Human ANA	368	9	−99.59 (10.31) **	−0.01 (0.03)	0.05 (0.01) **	0.005
Figure 8	Canine LD	Deer Mortality	280	77	80.17 (94.77)	44.83 (15.41) **	−0.04 (0.05)	<0.001
Figure 9	Canine ANA	Canine EHR	280	77	−491.84 (594.81)	12.23 (4.83) **	0.24 (0.29)	<0.001
Figure 8 and Figure 9	Canine EHR	Deer Mortality	280	77	923 (151.7) **	71.73 (18.57) **	−0.46 (0.08) **	<0.001
Figure 6 and Figure 7	Human EHR	Deer Mortality	368	92	24796.68 (10898.62) **	44.32 (16.22) **	−12.29 (5.4) **	<0.001
Figure 3 and Figure 4	Bb+	Canine EHR	280	77	−0.76 (0.76)	18.98 (3.44) **	--	<0.001
Figure 3 and Figure 4	Bb+	Human EHR	368	92	−0.04 (0.01) **	0.62 (0.2) **	--	<0.001
Figure 3 and Figure 4	Bb+	Human SFR	368	92	−0.05 (0.02) **	−0.54 (0.21) **	--	<0.001

**Table 7:** Summary of Deer Mortality and TBD Rates Hotspots by DMU. TBD = TickBorne Disease; DMU = Deer Management Unit; Bb = *Borrelia burgdorferi*; LD = Lyme Disease; EHR = Ehrlichiosis; SFR = Spotted Fever Rickettsiosis; ANA = Anaplasmosis.

Hotspots by DMU	Deer Mortality	Bb+	Human LD	Human SFR	Human EHR	Human ANA	Canine LD	Canine EHR	Canine ANA
Northeast	2703	O	-	-	-	-	-	-	-
South	1945	-	-	O	O	-	-	O	-
Muscatatuck & Dearborn	1855 and 1791	O	O	-	-	-	O	-	-
Northwest	1785	-	O	-	-	-	O	-	O
Wabash Valley	1759	-	O	-	-	-	O	-	O

## Data Availability

Data available upon request.
